# Eye care in rural communities: reaching the unreached in Sunderbans

**Published:** 2016

**Authors:** Sameera Ahmed

**Affiliations:** Programme Officer: Sightsavers, Kolkata, India.

**Figure:**
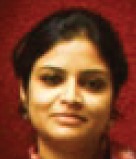
Sameera Ahmed

Good health is dependent on having services which are accessible and affordable. The Sunderbans in West Bengal, India, is a conglomeration of 106 islands, 52 of which are inhabited, and the remaining of which are home to India's famous tigers and the world's biggest mangroves. Small boats are the main mode of transport.

Sunderbans has the most deprived and marginalised population in India, with more than 40% of households living below the poverty line and 13% officially declared as the poorest of the poor.^1^

The health infrastructure is dismal and nearly 70% of health services in the Sunderbans is provided by the government (in the public health system). There is a huge shortfall in the number of primary health centres and in the number of people working in the public health system. The general morbidity rate is higher in Sunderbans than in the rest of West Bengal: about half of the children under 5 years are chronically malnourished and women are very vulnerable. The Sunderbans are prone to natural disasters, which further accentuates poor health conditions.

People in the Sunderbans are significantly disadvantaged in terms of eye health: whereas the prevalence of blindness amongst people aged 50 and above in West Bengal is 1.19% (and in India 1.0%), the prevalence of blindness in Sunderbans is 2.1%.

To address these challenges, Sightsavers is implementing the *Sunderbans Eye Health Service Strengthening Project* supported by Seeing is Believing. The five-year project, which started in September 2013, has recognised that multiple efforts and approaches are needed to address the inequity in eye health experienced by the people of Sunderbans. They have therefore employed various strategies to provide services and access to these remote communities: both by strengthening the existing channels of eye care provision and by creating new ones to provide eye health services in this rural part of West Bengal.

## Establishing vision centres

At the core of the project are 17 vision centres at locations where the ‘block’ (or local) government administration has its offices. Refraction, detection of cataract, provision of subsidised spectacles and making appropriate referrals are amongst the services delivered at these centres. People from the local area manage the vision centres after receiving specialised training to ensure a good quality service. The vision centres are linked to hospitals which help patients to make use of free and subsidised services that are available under government schemes, including operations. They are designed within a sustainability framework to ensure delivery of services beyond the life of the project.

**Figure:**
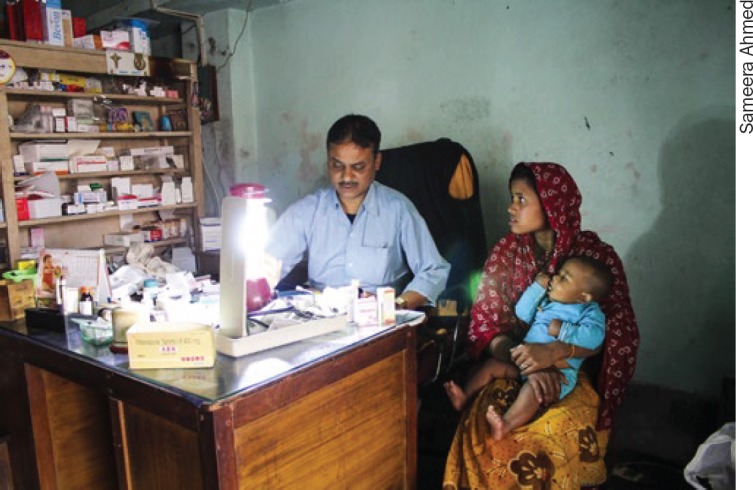
Rural Medical Practitioner Narayan Haldar at his clinic with a patient in Basanti, Sunderbans. INDIA

## Direct service delivery

As the vision centres gain popularity and acceptance, outreach camps are being held to reach even more remote areas and to focus on the needs of those who get left behind or are more vulnerable, including women and girls, older people, and people with disabilities. Vision assessment and outreach refraction services are also organised in schools, and the aim is to reach 457,000 children. Every child identified with correctable refractive error is offered free spectacles.

## Strengthening existing systems

It would be futile to address issues of accessibility while working independently of the government. At present, the sub-divisional government hospitals in Sunderbans are poorly managed, conducting fewer than one hundred cataract operations every year. The project is planning to conduct a facility survey to find out what needs to be done, and where, to enhance the capacity of these hospitals, for example by providing training in cataract management. In addition, 930 government health workers such as accredited social health activists (ASHA) and auxiliary nurse midwives (ANMs) are being trained to help with identifying cataract and creating awareness. The idea is to come alongside the hospitals and work with them to improve services.

While working in the community, one cannot ignore informal health seeking channels. One such channel in Sunderbans is rural medical practitioners (RMPs) or ‘village doctors’. They practise modern medicine without any registration or qualification. However, 62% of outpatients are treated by them.^1^ The project therefore aims to train 2,520 RMPs in primary eye care and appropriate referral.

## Creating new workers and awareness generation

Volunteers from villages are trained to improve eye health awareness and uptake of services locally. They are selected from the groups who are most likely to need eye care: women and young people.

The volunteers are called ‘Health Ambassadors.’ Through them, the project receives credibility, acceptance and reach. A total of 3,814 volunteers will be trained.

Service availability is not enough: people must be aware of services and be motivated to come forward. Through media such as radio, leaflets, posters, local folk theatre and interactive games the project is trying to make people aware that the service is now available – and that it is accessible and affordable.

## Monitoring using GIS technology

Monitoring effectiveness and reach is crucial. With a population as widely-dispersed as in the Sunderbans, the use of geographic information system (GIS) technology is indispensable. It has allowed us to record information from the baseline findings (about the need for eye care) on a map. Patient information from vision centres is then plotted on the same map so we can estimate coverage and see how far into the rural areas the vision centres reach. This is helping to ensure good coverage in all respects.
